# The impact of COVID–19 lockdown on dengue transmission in Sri Lanka; A natural experiment for understanding the influence of human mobility

**DOI:** 10.1371/journal.pntd.0009420

**Published:** 2021-06-10

**Authors:** Prasad Liyanage, Joacim Rocklöv, Hasitha Aravinda Tissera

**Affiliations:** 1 Department of Epidemiology and Global Health, Umeå University, Umeå, Sweden; 2 Ministry of Health, Colombo, 01000, Sri Lanka; 3 Department of Public Health and Clinical Medicine, Section of Sustainable Health, Umeå University, Umeå, Sweden; Mahidol Univ, Fac Trop Med, THAILAND

## Abstract

**Background:**

Dengue is one of the major public health problems in Sri Lanka. Its outbreak pattern depends on a multitude of drivers, including human mobility. Here we evaluate the impact of COVID–19 related mobility restriction (lockdown) on the risk of dengue in Sri Lanka.

**Methodology:**

Two-stage hierarchical models were fitted using an interrupted time-series design based on the notified dengue cases, January 2015 to July 2020. In the first stage model, the district level impact was estimated using quasi-Poisson regression models while accounting for temporal trends. Estimates were pooled at zonal and national levels in the second stage model using meta-analysis. The influence of the extended period of school closure on dengue in children in the western province was compared to adults.

**Findings:**

Statistically significant and homogeneous reduction of dengue risk was observed at all levels during the lockdown. Overall an 88% reduction in risk (RR 0.12; 95% CI from 0.08 to 0.17) was observed at the national level. The highest impact was observed among children aged less than 19 years showing a 92% reduction (RR 0.8; 95% CI from 0.03 to 0.25). We observed higher impact in the dry zone having 91% reduction (RR 0.09; 95% CI from 0.05 to 0.15) compared to wet zone showing 83% reduction (RR 0.17; 95% CI from 0.09 to 0.30). There was no indication that the overall health-seeking behaviour for dengue had a substantial influence on these estimates.

**Significance:**

This study offers a broad understanding of the change in risk of dengue during the COVID–19 pandemic and associated mobility restrictions in Sri Lanka. The analysis using the mobility restrictions as a natural experiment suggests mobility patterns to be a very important driver of dengue transmission.

## Author summary

Every year about 100 million symptomatic dengue cases occur with the highest-burden reported in Southeast Asia followed by Latin America. The burden is increasing and considered driven by population densities, human mobility, vector proliferation, and climate variability. Evaluation of the impact of human mobility on dengue transmission is methodologically challenging, but an important aspect in public health research.

The ongoing COVID-19 pandemic has resulted in dramatic reductions in human mobility due to social distancing measures. These population-level interventions in most countries have changed the exposure patterns to *Aedes* vectors and the spread of the dengue virus. The mobility restrictions due to the COVID-19 response in Sri Lanka provided a natural experiment to understand the importance of human mobility on dengue in an endemic setting.

Using interrupted time series design, we evaluated the impact of mobility restriction on dengue transmission in all 25 administrative districts, climate zones, and age groups in Sri Lanka. The results of this empirical study provided evidence of an overall statistically significant reduction of dengue during the period of mobility restriction in the country, which appears not to be related to changes in health seeking behaviour. Our study provides a methodological framework to quantify the impact of lockdown interventions on dengue transmission and its heterogeneous nature at different geographical, climatic, and socio-demographic levels within and between countries.

## Introduction

Dengue epidemics have become a serious concern with increasing morbidity and mortality in tropical and sub-tropical regions globally [1]. It is estimated that approximately 390 million new dengue infections and about 14,000 to 20,000 deaths occur annually in 128 dengue-endemic countries [2,3]. During the COVID-19 pandemic, a surge in dengue cases was reported in several low and middle income tropical and subtropical countries both in Asia and the Americas [4,5]. Thus, the combination of both COVID-19 and dengue outbreaks can have devastating consequences in affected communities and already stretched health care services [6].

Sri Lanka, a tropical island in the Indian Ocean, with a population of 21 million, has reported dengue cases since the early 1960s [7,8]. From 1991 to 2008, for almost two decades, dengue epidemics were reported once every two to three years on the background of endemic transmission [9]. A disproportionately high epidemic occurred in 2009, comprising 35,008 cases and 346 deaths (case–fatality rate 1%). From 2010 to 2016, dengue became a major public health problem, with a steady increase in cases from 28,473 to 55,150 cases in 2011 and 2016 respectively [10]. In 2017, Sri Lanka reported 186,101 dengue cases and 440 deaths (case fatality rate 0.24%), the highest number of cases reported in one year since dengue was incorporated into the integrated disease surveillance system as a notifiable disease in 1996 [11]. In 2019, a total of 104,695 cases with 157 deaths (case fatality rate 0.15%) were reported [12]. Annually, two dengue epidemics are reported in Sri Lanka of which the geographical distribution follows the temporal and spatial pattern of monsoonal rainfall. The major epidemic is seen in the wet zone which receives a relatively high mean annual rainfall of over 2,500 mm, particularly from the south–west monsoons from May to September. The dry zone receives a mean annual rainfall of less than 1,750 mm, mostly through the north-east monsoons, which extends from October to January and a distinct dry season from May to September [13].

However, in 2019, the mid-year peak in reported cases was shifted to the latter half of the year with most of the cases being reported during November and December. This trend continued into early 2020 with a total of 11,607 cases reported in January 2020 which is higher than the reported case (10,927) for the same period during the epidemic year 2017 [10]. Considering the high caseload reported in January it was expected for the May-September dengue season in 2020 also to be high. On the contrary, the reported number of dengue cases during April-June (second quarter) of 2020 dropped below the past 5-year national average. Due to the complex nature of the dengue transmission dynamics, the reasons for this observed historical island wide reduction should be interpreted with the careful analysis of the temporality of underlying drivers.

The government of Sri Lanka responded proactively to the global COVID-19 pandemic by imposing strict mobility restrictions throughout the country from March 2020 [14,15]. This strategy expanded from closing all schools since 12^th^ March, declaring public holidays and imposing quarantine curfew throughout the entire island since 20^th^ March [14]. People were encouraged to stay at home and a working from home policy was introduced. Inter-district movements were limited only to the essential services. Complete international travel restriction was implemented from 20^th^ March closing airports. After achieving acceptable control of COVID-19 transmission in the country, on 11^th^ May, the government decided to restore normalcy in life while maintaining quarantine curfew. The curfew was completely lifted from 28^th^ June 2020. Even during the curfew period, all health care institutions remained open and accessible to those who were in need.

The population-wide mobility restriction imposed as a response to the COVID-19 epidemic created an opportunity to evaluate the dengue epidemic before and after mobility restriction and estimate the counterfactual effect on human mobility on dengue transmission. Here we compared epidemiological characteristics of dengue transmission during the first (January to March) and second (April to June) quarters of 2020 with that of the averaged reporting of the preceding 5 years (2015–2019) in all districts in Sri Lanka. Further, we evaluated the impact of mobility restriction on dengue transmission patterns in all districts and dry zone, wet zone and national levels using the two-staged interrupted time series (ITS) method [16]. The impact of the extended period of school closure on dengue among children in the western province was compared to that of adults. We further investigated in the sensitivity analysis to identify whether there were any indications that the results could be confounded by changes in health seeking behaviour.

## Methods

Monthly dengue surveillance data for the study period January 2015 to July 2020 for each of the 25 administrative districts in the country were obtained from the National Communicable Disease Surveillance System [10]. The system captures symptomatic dengue patients according to a standard case definition of dengue [17]. Each dengue case is notified to the respective medical officer of health division in each district where the patient resides.

The mode and the timeframe of COVID-19 preventive interventions were obtained from the press release published on the official website of the Department of Government Information which was identified as the competent authority for circulation of COVID-19 related information to the public [14]. A time series of community mobility on the workplace and residential category were obtained from the COVID-19 community mobility reports freely shared by Google [18]. This mobility data was used to demonstrate deviations in the mobility patterns with respect to lockdown interventions and were not used in the statistical models.

### Statistical analysis

Analysis of the impact of lockdown on dengue across Sri Lanka was first assessed descriptively and then analytically using an ITS design. Stratified analysis was conducted to capture different seasonal transmission dynamics observed in the wet zone and the dry zone. The Western Province situated within the wet zone was considered as a separate entity due to the historically highest prevalence of dengue. The western province was further divided into two age strata i.e., age groups 0 to 19 years (children) and more than 20 years (adults) to capture the potential influence on the extended period of school closure on dengue transmission among children. Each stratum was then divided into a pre and post-lockdown period. The first quarter of the year 2020 (January, February and March) was considered as pre-lockdown period and the second quarter (April, May and June) was considered as post-lockdown period. This assumed any potential lockdown effects were delayed by two weeks from its onset in mid-March.

### Descriptive analysis

In the descriptive analysis, the five-year average (from 2015 to 2019) of the first and second quarters were taken as the comparator. The quarterly average of reported dengue cases per 100,000 population in each district was calculated to obtain quarterly expected (five-year average) and observed (the year 2020) values. Standardized incidence ratios (SIR) were calculated by dividing observed by expected dengue case counts in each quarter.

### Interrupted time series analysis

ITS analysis is a rigorous quasi-experimental research design for studying the effectiveness of such large-scale programs [16,19,20]. We made a nonlinear extension to the ITS design and estimated the impact of mobility restriction on dengue in each district before and after the intervention [16].

### First stage district-level analysis

We assumed a quasi-Poisson distribution and a logarithmic link function and used a Generalized Additive Modelling framework for the analysis [21]. The ITS models formulated as:

$$D_{it}∼quasiPoisson(\mu_{it})$$

$$log(E[D_{it}])=\alpha_{i}+\beta_{1i}(Year_{it})+\beta_{2i}(Month_{it})+\beta_{3i}(Lockdown_{it})$$

where *t* is time in months from January 2015 to July 2020; *D*_*it*_ is the aggregated monthly dengue cases from 2015 to 2020 in each *i* district at time t. α_i_ is the model intercept in each _i_ division. Year_it_ represents each year from 2015 to 2020. Month_it_ represents months from January to December in each year. The model was specified by fixed-effect variable for the year (Year_it_), and month (Month_it_) with the corresponding parameters (β_1i_) and (β_2i_) respectively to account for yearly variability, seasonality in risk and general time trends in dengue cases over the 67 months of the study period. The indicator variable for mobility, (Lockdown_it_) marked the onset of the lockdown leading to reduced mobility and was introduced as a binary dummy variable which set to zero during the pre-intervention period and to one during the lockdown period, i.e., April 2020 onwards. Estimates for the counterfactual scenario was obtained by setting the variable Lockdown_it_ to zero during the lockdown period. Relative risk along with a 95% confidence interval was calculated by exponentiation of the model coefficient (β_3i_) of the intervention (Lockdown_it_) variable. We developed the models by a rigorous investigation of all variables, including lagged effects, by model fit statistics, and model residual diagnostics (S1 Text).

### Second stage provincial, zonal and national level meta–analysis

The district-specific effect estimates for the mobility restriction were pooled at the meta-level to obtain joint estimates for the Western Province, the wet zone, the dry zone, and the national level. We applied multivariate fixed-effect meta-analysis using the *mvmeta* package in R for the meta-analysis [22]. Age-specific effect for children and adults in Colombo, Gampaha and Kalutara districts was meta-analysed to estimate the joint effect in the western province. Heterogeneity in exposure-response associations in administrative districts was assessed using the Cochran Q-test of heterogeneity [23].

### Sensitivity analysis

We conducted a sensitivity analysis by extending the ITS model including climate variables to adjust for any potential confounding effect of rainfall and temperature on the dengue transmission during the period of extensive mobility restriction. Depending on the availability of climate data, we selected Kalutara district in the Western Province for this adjustment. The same two-stage modelling procedure was applied using the ITS model; the first stage at sub-district (medical officer of health) level and the second stage meta-analysis at the district level. We used weekly dengue and climate data from 2010 for the analysis. The model was specified by a spline function for rainfall lag strata up to 12 weeks (Rain–lag 5 to 8 and Rain–lag 9 to 12), temperature lag up to 12 weeks (Temperature–lag 5 to 8 and Temperature–lag 9 to 12). The selected lag dimensions for climate variables in the models were based on our findings for the same study area using distributed lag non–linear models in a previous study conducted in the same district [24].

The sensitivity analysis was further extended to evaluate the potential impact of lockdown interventions on the health care seeking behaviour in the Kalutara district. We calculated the SIR for total hospital admissions (all-cause) and for other main communicable diseases in the district for the same study period. Data on non-severe dengue (dengue fever) and severe dengue (dengue haemorrhagic fever) was extracted from one of the hospitals in Kalutara district to investigate the potential change in seeking health care for milder dengue. The ratio between dengue fever and dengue haemorrhagic fever for each year from 2015 to 2020 was calculated and compared as a proxy for the direct impact of lockdown on dengue admissions. A detailed description of the climate adjustment and the evaluation of the impact of lockdown intervention on health care seeking behaviour is given in the supporting information (S2 Text).

## Results

A total of 451,299 suspected dengue cases were reported for the study period. The wet zone which includes nine districts, Colombo, Gampaha, Kalutara, Kandy, Kegalla, Nuwara-Eliya, Ratnapura, Galle and Matara reported 301,944 (66.9%) cases. The dry zone which has thirteen districts (Jaffna, Kilinochchi, Mulativu, Mannar, Vavuniya, Trincomalee, Anuradhapura, Polonnaruwa, Batticaloa, Ampara, Hambantota, Moneragala, Puttalam) reported 110,834 (24.6%) cases. The rest of the cases (38,521; 8.5%) were reported from the intermediate zone (Badulla, Matale, Kurunegala). Out of nine provinces in the country, the majority (171,784; 38.0%) of dengue cases were reported from the Western province which is the most populous province. The Western province is situated in the wet zone and has three districts; Colombo (the main metropolitan area in Sri Lanka), Gampaha, and Kalutara.

**Fig 1:**
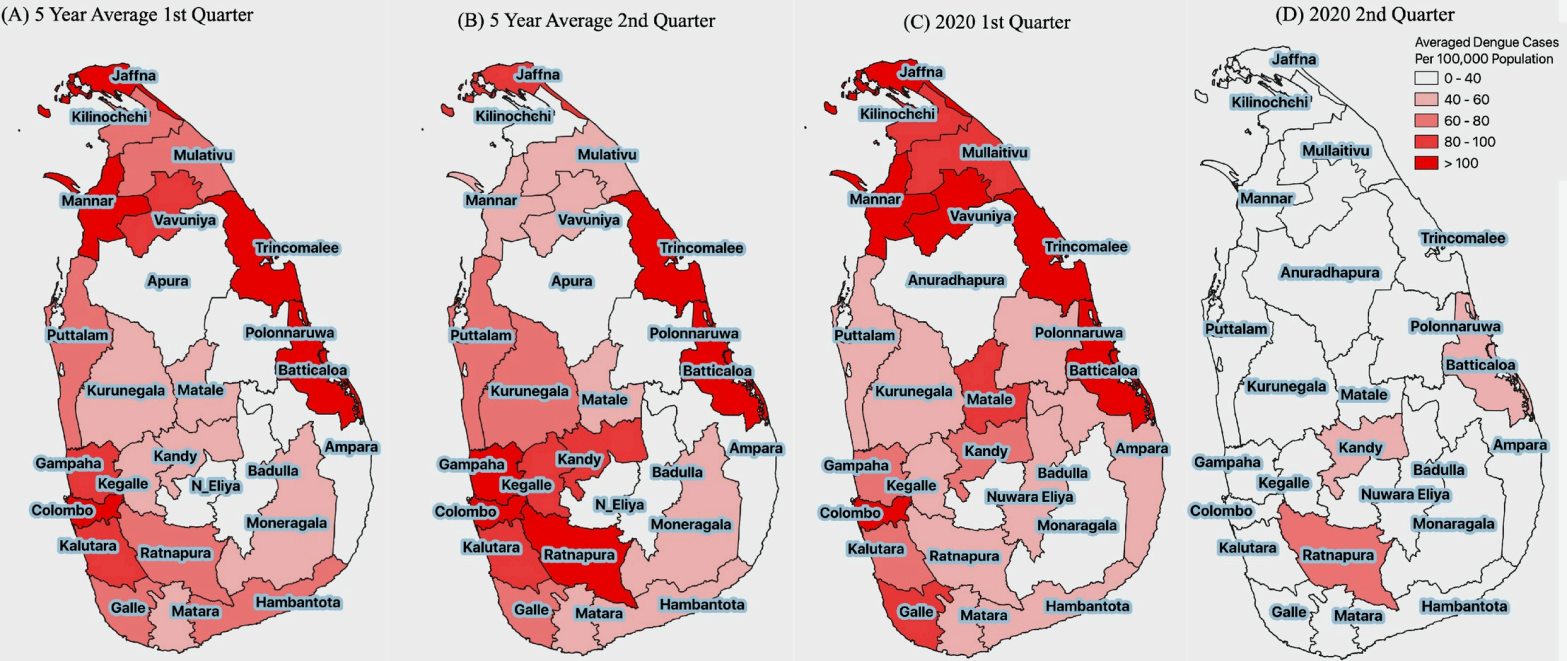
Distribution of dengue cases per 100,000 population during first and second quarters within administrative districts in Sri Lanka. (A) Averaged dengue cases from 2015 during 1^st^ quarter. (B) Five-year average during and 2^nd^ quarter. (C) Observed cases during first quarter 2020. (D) Observed cases during second quarter 2020 per 100,000 population in Sri Lanka. Source of the base file: https://data.humdata.org/dataset/sri-lanka-administrative-levels-0-4-boundaries.

The spatial distribution of averaged dengue cases for the past five years appeared to follow the monsoonal rainfall pattern. During the first quarter, the districts in the dry zone, which were usually getting rainfall from northeast monsoon from October to February had shown comparatively more dengue cases per 100,000 population. For the second quarter, the wet zone had reported more cases following the southwest monsoon during May to September. Noticeably, the reported cases were prominent in the Western province for both the 1^st^ and 2^nd^ quarters from 2015 to 2019. In the first quarter of 2020, more than the average number of dengue cases were reported predominantly among several districts in the dry zone. However, following the COVID-19 lockdown from mid-March, there was a ubiquitous island-wide reduction of dengue cases in the second quarter of 2020 (Fig 1).

With the implementation of mobility restriction, the Google mobility data shows a clear reduction in workplace mobility and increased residential mobility simultaneous to imposing island-wide quarantine curfew from 20^th^ March to 11^th^ May 2020. The mobility pattern gradually converged towards baseline once the country reverted to normalcy by 28^th^ June. The community mobility report was generated based on data from users who have opted into location history for their Google Account, so that the data may or may not represent the exact behaviour of a wider population. Even though it was not well represented by the Google mobility pattern, the schools and day-care centers were closed from 12^th^ March to July 2020 further restricting the mobility of children below 19 years.

**Fig 2:**
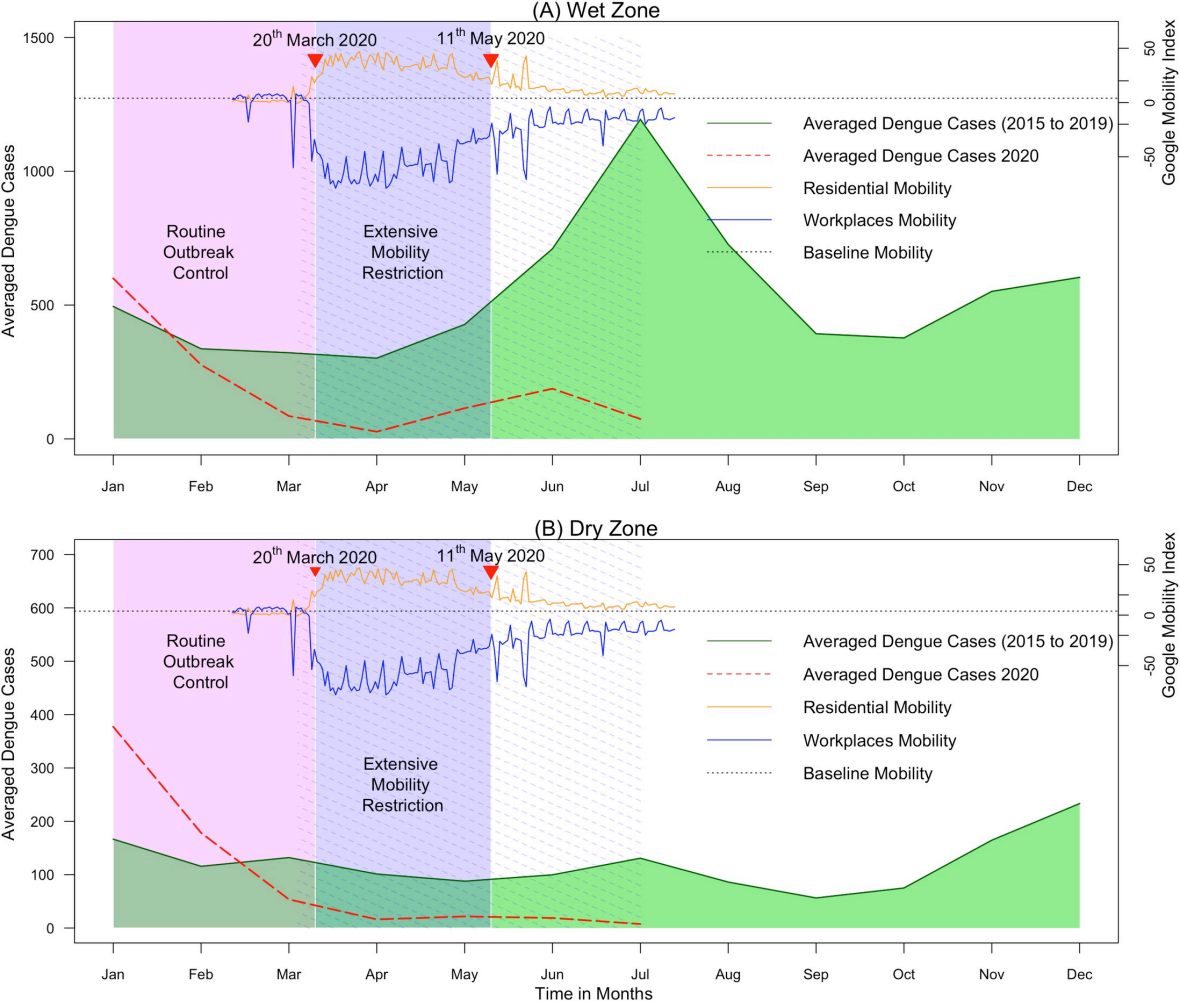
Reported dengue cases during 2020 compared to monthly averaged dengue cases reported from 2015 to 2019 averaged across all districts in climate zones in Sir Lanka. (A) Wet zone. (B) Dry zone. The green solid line shows the five-year average. Red dashed line shows cases reported during 2020. The purple solid area indicates the period of extensive mobility restriction; the purple dashed area indicates the period of school closure; the pink area indicates the period of routine dengue outbreak control interventions. The residential mobility represents the percentage change of daily mobility compared to the baseline residential mobility; the workplace mobility represents the percentage change in daily mobility compared to the baseline workplace mobility.

In the wet zone, the average monthly reported dengue cases per district for the year 2020 started just above the five-year average in January and declined to cross the five-year average during February (Fig 2A). Thereafter, during the period of extensive mobility restriction, it remained low reaching a nadir for the month of April. Though there was a slight increase number of cases during June, the expected seasonal peak for the month of July was not observed and further reduced compared to the reported number of cases in June. The SIR reduced from 1.01 to 0.26 (3.9–fold reduction) from the first to second quarter where the mobility restriction was imposed (Table 1).

**Table 1:** Quarterly distribution of dengue incidence and the relative risk estimates by national, zonal levels and by age groups in the western province in Sri Lanka. Quarterly distribution of dengue cases per 100,000 population in the first (January to March) and the second (April to June) quarter in 2020 compared to that of five-year average (2015 to 2019). The average values are presented with their respective standard deviation (SD). Standardized incidence ratio (SIR) and meta estimated relative risk with 95% confidence interval are presented.

	5–year average (1st Quarter)	5–year average (2nd Quarter)	2020 (1st Quarter)	2020 (2nd Quarter)	SIR Q1	SIR Q2	Relative risk (95% CI)
National	103.1(SD = 114.8)	82.1(SD = 57.4)	129.2(SD = 173.2)	18.1(SD = 18.1)	1.23	0.20	0.12 (0.08 to 0.17)
Dry zone	105.6 (SD = 157.0)	77.4 (SD = 65.8)	193.5 (SD = 238.4)	13.6 (SD = 18.4)	1.55	0.15	0.09 (0.05 to 0.15)
Wet Zone	72.9 (SD = 45.1)	94.6 (SD = 46.2)	64.8 (SD = 29.6)	24.6 (SD = 21.0)	1.01	0.26	0.17 (0.09 to 0.30)
Western Province	118.2 (SD = 50.1)	135.1(SD = 39.4)	87.3(SD = 24.9)	22.7 (SD = 9.9)	0.77	0.19	0.16 (0.06 to 0.43)
Children	155.8 (SD = 118.1)	214.1 (SD = 119.7)	131.9 (SD = 86.2)	17.2 (SD = 6.5)	0.85	0.08	0.08 (0.03 to 0.25)
Adults	101.9 (SD = 36.4)	156.5 (SD = 46.4)	95.7 (SD = 33.3)	29.0 (SD = 9.9)	0.94	0.19	0.14 (0.05 to 0.35)

**Fig 3:**
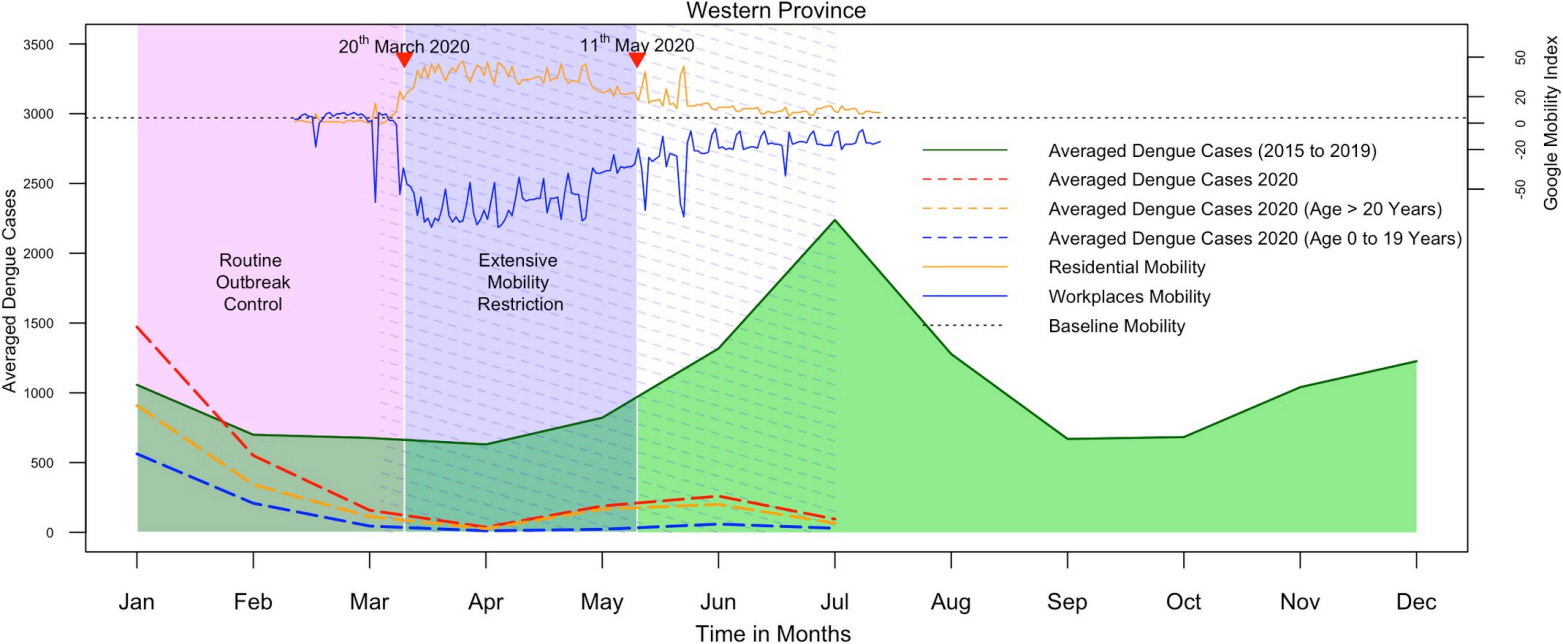
Reported dengue cases among children and adults during 2020 compared to monthly averaged dengue cases reported from 2015 to 2019 averaged across all districts in the western province in Sir Lanka. The green solid line shows the five-year average. Red dashed line shows cases reported during 2020.The blue and orange dashed lines indicate the monthly average number of dengue cases among children (age 0 to 19 years) and adults (age more than 20 years) reported respectively in 2020. The purple solid area indicates the period of extensive mobility restriction; the purple dashed area indicates the period of school closure; the pink area indicates the period of routine dengue outbreak control interventions. The residential mobility represents the percentage change of daily mobility compared to the baseline residential mobility; the workplace mobility represents the percentage change in daily mobility compared to the baseline workplace mobility.

In the dry zone, the epidemic curve for 2020 started well above five-year average and declining during March. Thereafter, continued to remain low until it reached the lowest value during July (Fig 2B). The SIR was reduced from 1.55 to 0.15 (10.5–fold reduction) from the first to the second quarter. Further, the SIR value observed for the first quarter in the dry zone (1.55) was 1.5–fold higher compared to that of the wet zone (1.01) suggesting a larger contribution of the dry zone to the dengue epidemic observed in early 2020. The western province followed a similar pattern to the wet zone (Fig 3). The SIR reduction for the age group between 0 to 19 years was remarkably higher (10.6 fold) compared to that of adults (4.9 fold).

Zonal and provincial level meta estimates of the interrupted time series analysis show that there was a statistically significant impact of mobility restriction on the reported dengue cases in national, zonal and provincial levels (Table 1). Overall a 88% reduction in dengue (RR 0.12; 95% CI from 0.08 to 0.17) was observed at the national level. The highest impact was observed among children (age 0 to 19) having a 92% reduction (relative risk of 0.08 with 95% confidence interval; 0.03 to 0.25). The impact in the dry zone (91% reduction; RR 0.09; 95% CI from 0.05 to 0.15) was more than on the wet zone (83% reduction; RR 0.17; 95% CI from 0.09 to 0.30). Additional analysis conducted for the age group of 20 to 29 years showed RR of 0.12 (95% CI 0.04 to 0.32). S1 Table shows the quarterly distribution of reported dengue incidence per 100,000 population in each district in Sri Lanka and the respective SIR. Except for Matara in the wet zone and Jaffna, Mannar and Monaragala districts in the dry zone, all other districts showed statistically significant impact (S2 Table). Cochran Q test of heterogeneity was not statistically significant at a p-value of 0.05 for any of the levels analyzed.

**Fig 4:**
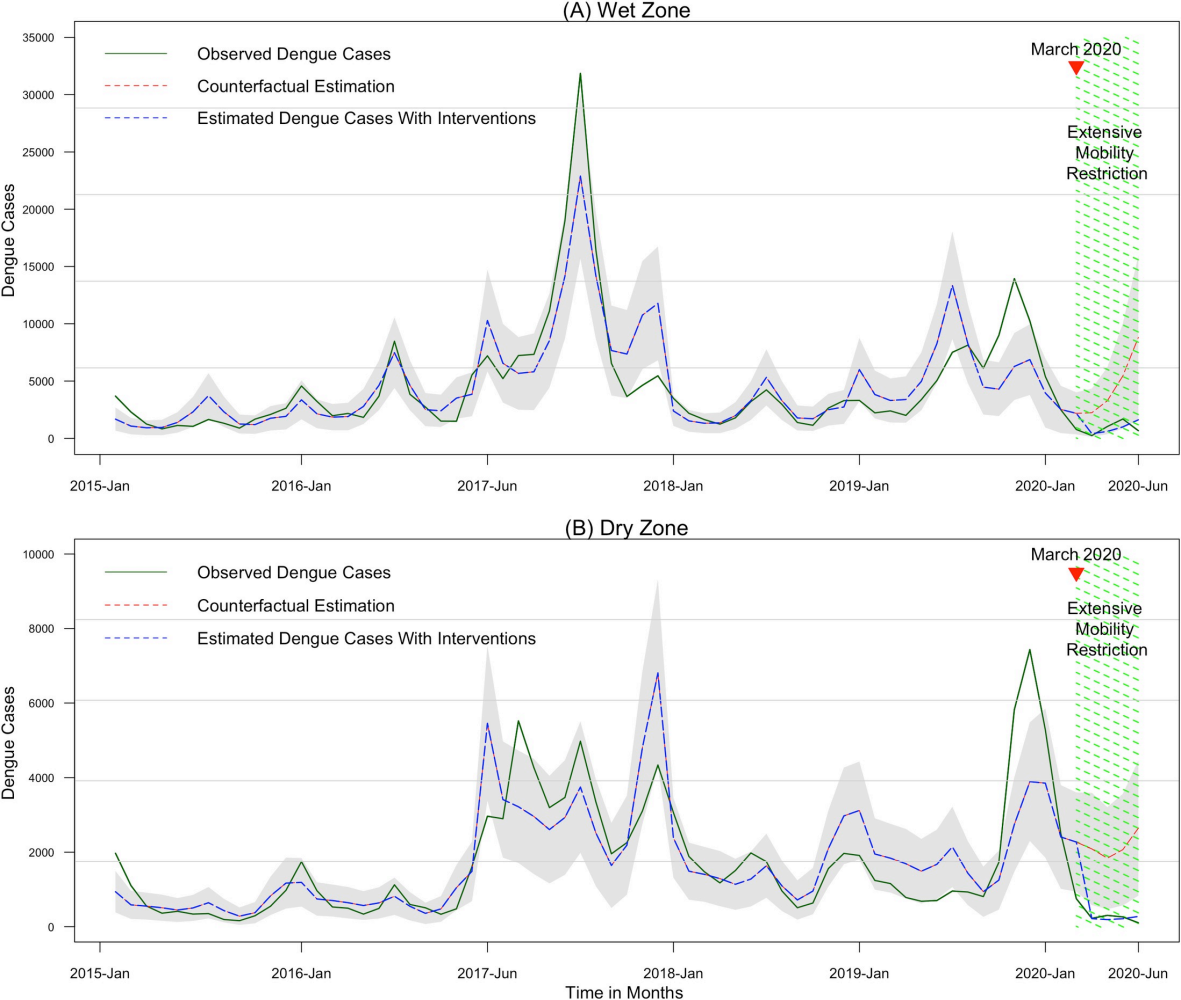
Time series plot of observed and estimated dengue cases from 2015 to 2020 July in wet and dry zones in Sri Lanka. (A) Wet zone. (B) Dry zone. The green line represents observed dengue cases. Blue dashed line represents the model predicted or estimated dengue cases with the lockdown intervention in place. The red dashed line represents estimated dengue cases without intervention or the counterfactual estimation. The grey zone indicates the lower and upper limits of the 95% confidence intervals for estimated dengue cases at each month.

The time series plots show the counterfactual predictions in wet and dry zones in Sri Lanka (Fig 4). In both zones, the observed dengue cases showed a gradually increasing trend until it dropped down to the lowest level during period of mobility restriction in 2020. If mobility restriction had not taken place (counterfactual scenario) the models predict a higher number of dengue cases of (counterfactual estimates) for the COVID-19 intervention period in both zones.

The results of sensitivity analysis showed that the meta estimate obtained after adjusting the confounding effect of rainfall and temperature for the Kalutara district was 0.23 (95% confidence interval; 0.15 to 0.36). The estimate was not statistically different from the first stage model estimate of 0.25 (95% confidence interval; 0.08 to 0.74) obtained for Kalutara through the main analysis (Table A in S2 Text and S2 Table). The comparative analysis of hospital admission due to all health conditions and communicable diseases showed no strong indications that changes in health seeking behaviour could affect the estimates of the reduction in dengue during the lockdown. Further, sensitivity analysis revealed that the ratio of dengue fever to severe dengue fever (dengue haemorrhagic fever) from one hospital in the Kalutara region was within the same range in 2020 compared to earlier years. A detailed description of the sensitivity analysis is available in the S2 Text.

## Discussion

With the COVID-19 lockdown, Sri Lanka experienced its lowest reported dengue cases compared to the five-year average with considerable relief to the health system. Here in this study, we quantified the indirect effect of COVID-19 related social distancing interventions on the transmission of dengue in different climate zones and age groups in Sri Lanka. We found an overall 88% (RR 0.12; 95% CI from 0.08 to 0.17) reduction of dengue risk across the nation involving both wet and dry zones.

The simultaneous reduction of dengue risk in two climate zones with different transmission patterns coincided with the proactive COVID-19 preventive interventions. The government encouraged the people to stay at home from as early as 12^th^ March by closing schools and universities, banning public gatherings and imposing a curfew. As evident by the Google community mobility patterns, these social distancing measures dramatically reduced the mobility of the people outside the residence [18]. In Sri Lanka, a higher prevalence of vector breeding places was identified in out-of-home locations such as factories, construction sites, schools, religious and, public places (premise index on average 40 to 60%) compared to households (premise index on average 5 to 20%) [25–27].

During the lockdown period, the dengue outbreak control program conducted with civil–military partnership was interrupted. The public health staff and the military were heavily involved with COVID-19 mitigation activities with less emphasis on dengue source reduction [15]. However, the community engagement to actively remove breeding habitats in and around homes may have improved during an extended period spent at home during the lockdown. People were encouraged on home gardening by the government and social media and had more time to pay attention to the vector breeding in their premises [14]. The regular garbage and container removal activities through local authorities were undisrupted and continued in dengue high-risk urban areas throughout the entire lock–down period [28,29]. Deviation from the out of home locations especially during the daytime may have reduced the opportunities of human vector contact for the day-biting *Aedes* vectors [30]. Therefore, the restriction of the mobility to out-of-home locations where the dengue transmission potential was high along with the active engagement of the community on source reduction of their premises may have synergistically influenced the observed reduction of dengue incidence. Since human is acting as a vessel for the virus, the reduced human mobility may have limited the spread of the dengue virus from hot-spots to the other parts of the country.

The dry zone of Sri Lanka, with a larger landmass and having a lower population density showed a higher impact of mobility restriction (RR 0.09; 95% CI 0.05 to 0.15) compared to the wet zone (RR 0.17; 95% CI 0.09 to 0.30) and to the more urban western province which is a part of the wet zone (RR 0.16; 95% CI 0.06 to 0.43). It has been previously observed that secondary dengue outbreaks were initiated when infected people from endemic wet zone cross the district boundaries and travel into the dry zone areas. This phenomenon was significantly restricted during the 2020 strict lockdown period (e.g. migrant construction and apparel workers were restricted to their boarding places with no access to their home-towns). Low population densities, differences in the climate factors, distribution and type of vector breeding places, heard immunity to circulating virus and human mobility patterns in the dry zone compared to the wet zone may have contributed to observed sharp decline in the model predicted dengue cases with intervention in the dry zone. The impact of the community mobilization campaign as a continued behavioural response to a large dengue outbreak that occurred during the latter part of 2019 and extending to early 2020 in the dry zone may also have contributed. The local communities might have been motivated to actively remove common breeding places in and around dwellings during the extended homestay.

No public health intervention in the past had previously achieved a reduction of the dengue cases below 40 per 100,000 per quarter in the western province [10]. During the previous outbreaks, in the western province, the highest dengue incidence rate was observed among the 20–29 years age group followed by the 10–19 years age group. However, we found that the highest impact of mobility restriction was on the children belonging to the age group of 0 to 19 years (RR 0.08; 95% CI 0.03 to 0.25) as compared to the adults (RR 0.14; 95% CI 0.05 to 0.35) and in particular to the age group of 20–29 (RR 0.12; 95% CI 0.04 to 0.32). This observation strongly suggests that the prolonged period of school closure from 12^th^ March to 6^th^ July has had nearly 1.5 times the higher impact on the dengue incidence among children when compared to adults.

In Singapore, in 2020, the social distancing measures had an opposite impact causing an increased of over 37.2% in the dengue cases from the baseline [31]. It was highlighted in many areas of Singapore that residences were the most commonplace of mosquito activity rather than workplaces. Many employment-age adults in Singapore usually work in air-conditioned indoor environments which prevent exposure to the *Aedes* vectors during day time [32]. According to the dengue situation update by the World Health Organization, during 2020, the dengue activity showed decreasing trends in countries such as the Philippines (81% reduction), Laos, Malaysia and, Vietnam (64.8% reduction) compared to the same period in 2019 [33]. The heterogeneous pattern observed in different countries indicates that the indirect effect of mobility restriction on dengue depends on the extent to which the mobility restriction was implemented and contextual factors governing the varying degrees of location-specific human vector contact. Several studies conducted in the recent past also highlighted the importance of human movements for the spread of the dengue virus and its heterogeneous nature [34–36].

In Sri Lanka, an integrated surveillance system of communicable diseases includes dengue and has an Island wide coverage through trained and dedicated clinical and public health staff. National surveillance data are based on timely reports with a high yield capturing symptomatic dengue patients seeking medical care based on standard surveillance classification. Once detected cases are reported to the respective public health medical officer of the area where the patient resides. Dengue is a notifiable disease through the surveillance system since 1996. More recently, in 2010, a web-based early-warning sentinel surveillance system was established in the major hospitals across the country to augment the existing system. Despite the lock-down measures, both systems were operating with a weekly feedback mechanism in place as part of the regular monitoring and evaluation process. During the lockdown period, outpatient departments and inpatient care in all curative institutions were functioning without disruption. Community awareness on dengue prevention continued and early health-seeking for febrile patients was encouraged. Despite quarantine curfew in place, the sick people were allowed access to the hospitals round the clock. Mobile clinics were conducted particularly in strict lockdown areas with active COVID-19 clusters to attend to the acute health needs of people. National guidelines were issued by the Ministry of Health on managing dengue during COVID–19 epidemic [37]. If any decline in dengue cases had occurred due to reduced access to health, it cannot be explained by the continued reduction in dengue cases even after travel restrictions were lifted in May 2020. A substantial increase in the reported number of leptospirosis cases during the lockdown period indicates pervasive health care accessibility [12]. We have thoroughly evaluated the impact of mobility restriction on hospital admissions and specifically for dengue and other communicable diseases in one of the districts in the western province and presented in the supplementary material (S2 Text). There we found that, despite the lockdown interventions, a considerable number of hospital admissions were reported during the second quarter of 2020 from the curative institutions in the Kalutara district. This observation highlighted the unhindered access to health care facilitates. However, as expected, parallel to the mobility restriction, the lowest number of admissions were reported during the second quarter of 2020 reducing the SIR from 0.97 to 0.75 (1.3-fold reduction). This reduction was not only due to reduction of health care seeking behaviour itself but also due to the joint effect of concomitant reduction of admissions related to communicable and non-communicable diseases such as acute injuries and it includes the reduction of dengue [38,39]. The observed reduction of SIR of dengue was from 0.9 to 0.34 (2.6-fold reduction) for the same setting. This proportionately higher reduction further suggests altered dengue transmission dynamics with restricted human mobility. Further, if there had been an impact on the lockdown on health seeking behaviour of dengue patients, one would still expect severe dengue (dengue haemorrhagic fever) cases to seek care, while milder dengue cases (dengue fever) would perhaps go unnoticed. Our sensitivity analysis showed that the ratio of non-severe to severe dengue remained in the same range as before during the pandemic (S2 Text).

The non-availability of the climate information for all 25 districts at the time of this analysis could be one of the limitations. Due to the absence of climate information in the models, the confounding effect of climate may not be captured completely. However, our sensitivity analysis conducted with rainfall and temperature data in ten sub-district locations in Kalutara showed that the meta estimate (RR 0.23; 95% CI 0.15 to 0.36) which was not significantly different from the district estimate obtained for Kalutara in the first stage models (RR 0.25; 95% CI 0.08 to 0.74) without climate information. Furthermore, it was difficult to verify the vector abundance in relation to the observed reduction in the dengue cases as the door-to-door vector surveillance activities in and around high-risk localities also have been disrupted to some extent during the lock-down period in Sri Lanka.

Considering the findings of this unique natural experimental situation, we emphasize the importance of vector control in schools, workplaces, industrial zones, hospitals, and public places where people tend to gather more during the daytime when the vector is also active. Better understanding human mobility is an important contributor, early identification of the clusters and explores the possibility of identifying those who have frequent travel requirements to and from high-risk areas for selective vector and fever surveillance would be important. Promoting usage of the newly introduced “Dengue Free Child” app [40] enables early identification of children having fever and encourage them to stay at home while considering appropriate curative and preventive measures. Previous studies conducted in the district of Colombo have highlighted the presence of proportionately the highest prevalence of dengue vector breeding places in and around schools [27]. The vector control interventions in and around school premises should be enhanced with the active participation of the education department, parents and the community. In this context, multi-sector involvement, participatory approach in vector control policy with regular monitoring and supervision of the implementation at the schools would play an important role.

Our study offers a broader understanding of the epidemiology of dengue as a response to mobility restriction during the initial months of the COVID–19 pandemic in Sri Lanka. The finding highlights human mobility is a strong driver of the incidence and nationwide spread of dengue. The methodology can be used to explain heterogeneous outcomes of mobility restriction on dengue across regions and subgroups and its public health implications on prevention and control.

## Supporting information

S1 Text Evaluation of model fit characteristics. (DOCX)

S2 Text Sensitivity analysis. (DOC)

S1 Table Comparison between the quarterly distribution of dengue incidence by administrative districts. (DOCX)

S2 Table Impact of lockdown intervention by administrative districts as estimated by the ITS model. (DOCX)
