# Supplementary material for: The impact of COVID–19 lockdown on dengue transmission in Sri Lanka; A natural experiment for understanding the influence of human mobility
Source: PLoS Negl Trop Dis. 2021 Jun 10;15(6):e0009420. doi: 10.1371/journal.pntd.0009420 (PMC8192006; doi:10.1371/journal.pntd.0009420)
Supplement: S1 Text — (DOCX) [file pntd.0009420.s001.docx]

**Supporting Information (S1 Text)**

**Evaluation of model fit characteristics**

**Model fit statistics**

The final interrupted time series (ITS) regression model was selected after sequential fitting of temporal confounders and adjusting the lag value for the lockdown dummy variable. The best fit model was specified by fixed-effect variable for year and month to account for yearly variability and seasonality. The lockdown dummy variable with a lag of zero showed the best fit. Table A shows the adjusted R square, deviance explained, and p-value for each district obtained using the best fit model at the first stage division-specific analysis.

**Table A: Model fit statistics of the best fit models by districts**. Percentage deviance explained, adjusted R square, and corresponding p values for the coefficient obtained in the first stage models are demonstrated.

|  | **Deviance explained (%)** | **Adjusted R square** | **p-value for the model coefficient** |
| --- | --- | --- | --- |
| Ampara | 82·63 | 0·76 | 0·000 |
| Anuradhapura | 74·60 | 0·65 | 0·010 |
| Badulla | 73·34 | 0·54 | 0·015 |
| Batticaloa | 72·04 | 0·51 | 0·002 |
| Colombo | 69·64 | 0·53 | 0·026 |
| Galle | 78·10 | 0·68 | 0·002 |
| Gampaha | 78·25 | 0·68 | 0·025 |
| Hambantota | 84·03 | 0·79 | 0·005 |
| Jaffna | 77·08 | 0·64 | 0·107 |
| Kalmunai | 78·90 | 0·64 | 0·033 |
| Kalutara | 78·61 | 0·71 | 0·016 |
| Kandy | 73·86 | 0·57 | 0·044 |
| Kegalle | 83·23 | 0·81 | 0·041 |
| Kilinochchi | 65·24 | 0·35 | 0·050 |
| Kurunegala | 80·62 | 0·76 | 0·014 |
| Mannar | 53·17 | 0·24 | 0·195 |
| Matale | 59·40 | 0·32 | 0·049 |
| Matara | 86·02 | 0·80 | 0·311 |
| MC Colombo | 60·63 | 0·39 | 0·071 |
| Moneragala | 85·13 | 0·82 | 1·000 |
| Mullaitivu | 64·09 | 0·45 | 0·026 |
| Nuwara Eliya | 72·18 | 0·62 | 0·004 |
| Polonnaruwa | 74·58 | 0·67 | 0·001 |
| Puttalam | 77·38 | 0·72 | 0·094 |
| Ratnapura | 82·97 | 0·80 | 0·118 |
| Trincomalee | 66·51 | 0·26 | 0·054 |
| Vavuniya | 65·63 | 0·41 | 0·035 |

**Model residual diagnosis:**

Figures A and B show the residual analysis and the auto-correlation of the selected model for the national-level analysis. The straight diagonal line in the Normal Q-Q plot and the histogram of residuals suggest approximately a normal distribution for residuals. We find a similar random distribution of residual versus the linear predictor and the response. Figures A and B suggest acceptable autocorrelation of residuals as the total number of residual lags deviated less than 2SD in the autocorrelation plot and, thus, follows the assumption of normal random distribution.


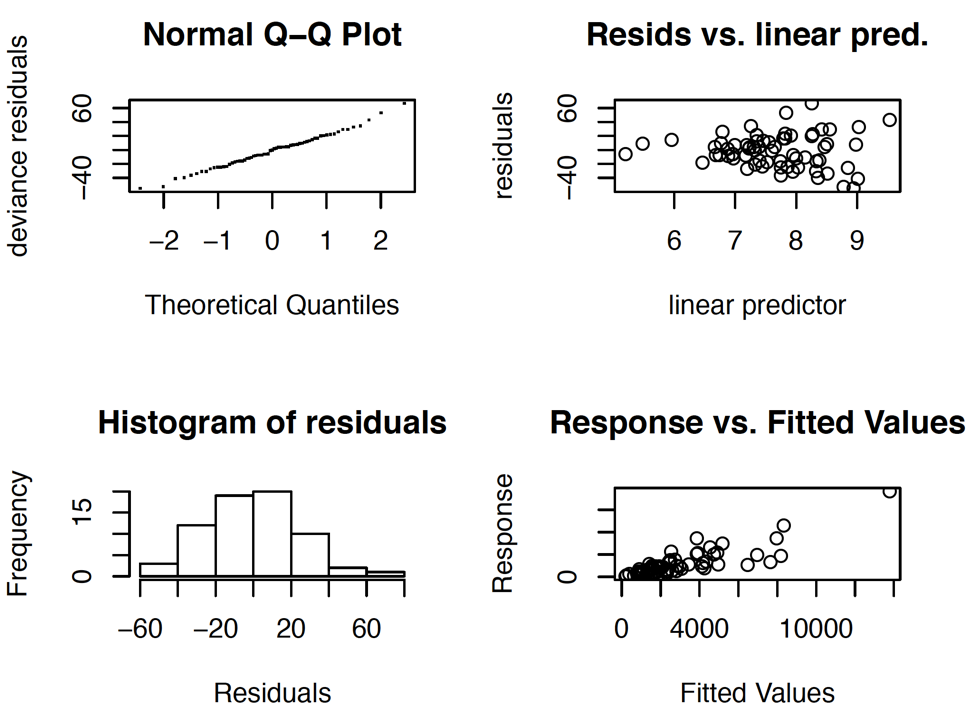


**Fig A. Residual analysis of the selected model.** The Normal Q-Q plot (upper left panel) shows the residual deviance in relation to theoretical quintiles in the model. The upper right panel shows the Residual versus Linear Predictor. The lower left panel shows the Histogram of Residuals and the lower right panel shows the Response versus Fitted values.

**
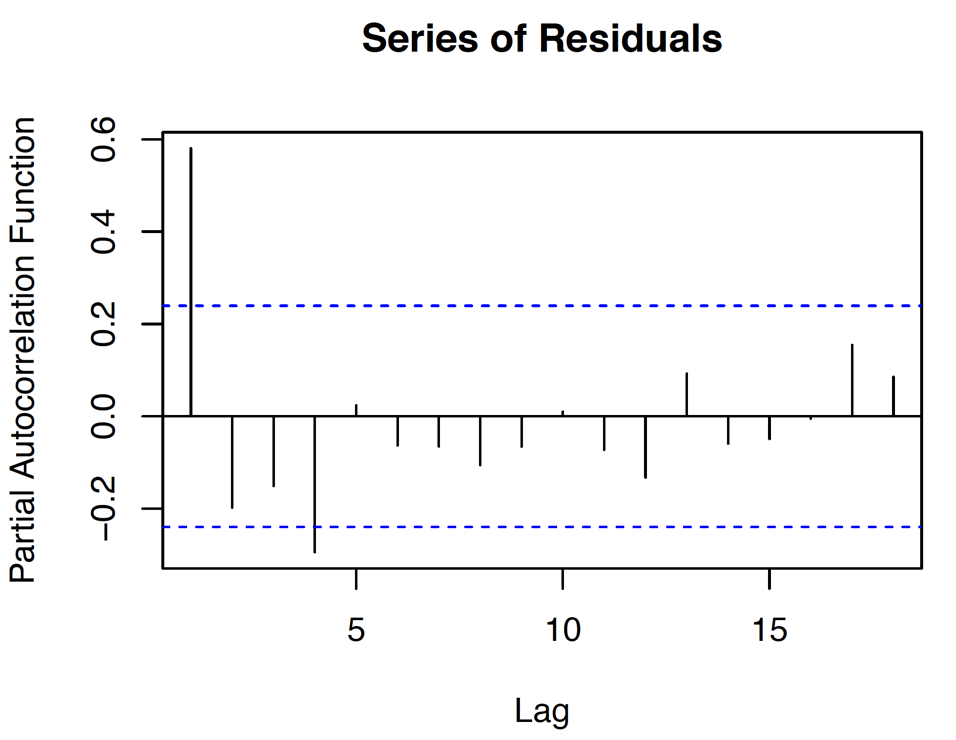
**

**Fig B. The partial Autocorrelation function of residuals.** The x-axis shows the lag period and Y-axis shows the partial autocorrelation function. The blue dotted horizontal lines represent the upper and lower limits of 2 standard deviations of the autocorrelation function.
