# Supplementary material for: The impact of COVID–19 lockdown on dengue transmission in Sri Lanka; A natural experiment for understanding the influence of human mobility
Source: PLoS Negl Trop Dis. 2021 Jun 10;15(6):e0009420. doi: 10.1371/journal.pntd.0009420 (PMC8192006; doi:10.1371/journal.pntd.0009420)
Supplement: S2 Text — (DOC) [file pntd.0009420.s002.doc]

Supporting Information (S2 Text)

**Sensitivity Analysis**

**Adjustment for the climate variables using Interrupted Time Series analysis in Kalutara district.**

Kalutara district was selected as it is situated in the highly dengue-endemic western province of the country and the lagged effect of climate variables on dengue is already quantified [1]. It is situated in the western province adjacent to the southern border of Colombo, the main metropolitan area in the country. The geographical boundaries of the district fall within the latitudes 6°19'N – 6°49'N and longitudes 79°53'E – 80°22'E. It expands from the coastal region in the west to the edge of mountain ranges and rainforests in the central part of the island. Altitude is below 150 m in most parts. Administratively, the district is divided into ten medical officer of health (MOH) divisions.

We extracted weekly notified dengue cases through the national integrated communicable disease surveillance system. Daily rainfall and temperature data from 2010 up to 20^th^ week 2020 were extracted from eighteen rainfall and three temperature monitoring stations within the district, which operate by the Department of Meteorology, Sri Lanka. We aggregated weekly cumulative rainfall and weekly means of maximum temperature using daily data.

**Statistical analysis:**

We made a nonlinear extension to the ITS design and estimated the impact of mobility restriction on dengue in each MOH division before and after the intervention [2]. We incorporated climate variables within the regression model to account for their confounding effect on dengue transmission. Two staged hierarchical analysis conducted; at MOH division level in the first stage and meta analysing at the district level in the second stage.

*First stage MOH division level analysis*

We assumed a quasi–Poisson distribution and a logarithmic link function and used a Generalized Additive Modelling framework for the analysis (3).^3^ The ITS models were formulated as:

*D_it_ ~ quasiPoisson(µ_it_)*

log(E[D_it_]) = α_i_ + β_1i_ (Rain_it-(5 to 8)_) + β_2i_ (Rain_it-(9 to 12)_) + β_3i_ (Temperature_it-(5 to 8)_) + β_4i_ (Temperature_it-(9 to 12)_) + β_5i_(Lockdown_it_) + time

where *t* is time in months from January 2015 to July 2020; *D_it_* is the aggregated monthly dengue cases from 2010 to 20^th^ week 2020 in each *i* MOH division at time t. The model was specified by smooth functions for climate variables (rainfall and maximum temperature) with respective lag strata selected based on the lag–exposure-response association quantified in the previous publication in the same setting [1]. β_1i_ and β_2i_ were used for rainfall lag strata five to eight weeks and nine to 12 weeks respectively. β_3i_ and β_4i_ were used for temperature lag strata five to eight weeks and nine to 12 weeks respectively. Time variable was included to account for the long-term trend. The indicator variable for mobility, Lockdown_it_, marked the onset of the lockdown leading to reduced mobility and was introduced as a binary dummy variable which set to zero during the pre-intervention period and to one during the lockdown period, i.e., 12^th^ week onwards. Estimates for the counterfactual scenario were obtained by setting the variable Lockdown_it_ to zero during the lockdown period. Relative risk along with a 95% confidence interval was calculated by exponentiation of the model coefficient β_5i_ of the intervention variable.

*Second stage district level meta-analysis:*

The MOH division-specific effect estimates for the mobility restriction analysed at the meta-level to obtain joint estimates for the Kalutara district. We used the *mvmeta* package in R for the meta–analysis [4]. ﻿Heterogeneity in exposure-response associations in MOH divisions was assessed using the Cochran Q–test of heterogeneity [5].

**Table A:** Relative risk with 95% confidence interval as estimated by the interrupted time series analysis after regressing the effect of rainfall and temperature for all medical officer of health divisions in Kalutara district in Sir Lanka.

|  | **Relative Risk** | **p value** |
| --- | --- | --- |
| Panadura | 0·10 (0·03 to 0·33) | 0·000 |
| Bandaragama | 0·08 (0·02 to 0·33) | 0·001 |
| Madurawala | 0·65 (0·33 to 1·29) | 0·221 |
| Horana | 0·50 (0·26 to 0·95) | 0·036 |
| Ingiriya | 0·19 (0·05 to 0·72) | 0·015 |
| Matugama | 0·14 (0·05 to 0·41) | 0·000 |
| Agalawatta | 0·15 (0·04 to 0·52) | 0·003 |
| Walallavita | 0·23 (0·06 to 0·93) | 0·040 |
| Bulathsinhala | 0·31 (0·16 to 0·61) | 0·001 |
| Palindanuwara | 0·04 (0·00 to 0·45) | 0·010 |
| **Meta estimate** | **0·23 (0·15 to 0·36)** | **0·000** |

The lockdown intervention showed a statistically significant impact on dengue even after adjusting for the confounding effect of climate variables in the Kalutara district (Table A). The joint estimate for the counterfactual effect on the dengue cases due to mobility restriction as estimated by meta analysing all divisional estimates is -1.4602 (-1.9094 to -1.0109). The overall relative risk is 0·23 (0·15 to 0·36). Cochran Q-test for heterogeneity was statistically not significant (p-value 0.258) suggesting homogeneous impact across all the districts.

**Evaluation of health care seeking behaviour**

We obtained monthly admissions to all curative institutions in Kalutara district from 2015 to 2020 second quarter from the indoor morbidity and mortality records available. Those institutions where the second-quarter data were not yet available were removed from the analysis. The reported dengue cases in each month from 2015 were deduced to adjust for any potential impact on the total number of admissions by the reduced number of dengue cases in the second quarter of 2020. We calculate the standardized incidence ratio (SIR) for the first and second quarter for the total number of hospital admissions and compared it with that of dengue incidence per 100,000 population. Table B compares the SIR for hospital admissions (calculated for 100,000 population) with that of reported dengue incidence and four other selected communicable diseases.

**Table B:** **Hospital admissions versus reported dengue, leptospirosis, chickenpox, tuberculosis, and dysentery incidence in the first and second quarters in Kalutara district.** All values were calculated for the 100,000 population.

| Disease | 5-year average  (1st Quarter) | 5-year average  (2nd Quarter) | 2020  (1st Quarter) | 2020  (2nd Quarter) | SIR Q1 | SIR Q2 |
| --- | --- | --- | --- | --- | --- | --- |
| Admissions | 4033.25 | 4254.13 | 3912.82 | 3181.08 | 0.97 | 0.75 |
| Dengue | 83.8 | 99.2 | 75.4 | 33.3 | 0.90 | 0.34 |
| Leptospirosis | 5.43 | 6.20 | 6.47 | 18.09 | 1.19 | 2.92 |
| Chickenpox | 8.13 | 7.07 | 9.82 | 6.47 | 1.21 | 0.91 |
| Tuberculosis | 6.73 | 4.06 | 4.58 | 5.40 | 0.68 | 1.33 |
| Dysentery | 1.29 | 1.41 | 0.25 | 0.00 | 0.19 | 0.00 |

Despite the lockdown interventions, a considerable number of admissions were reported during the second quarter of 2020 from the curative institutions in the Kalutara district. This observation highlighted the pervasive availability of health care facilities. As expected, parallel to the mobility restriction, the lowest number of admissions were reported during the second quarter of 2020 reducing the SIR from 0.97 to 0.75 (1.3-fold reduction). This reduction may not only be due to reduction of health care seeking behaviour itself but also due to the joint effect of concomitant reduction of admissions related to communicable and non-communicable diseases such as acute injuries (including the reduction in dengue). According to the annual health bulletin data, it is also seen that the majority of hospital admissions were due to non-communicable diseases [6]. Reduction of acute injuries including road traffic accidents may have contributed to the observed reduced number of hospital admissions (6). According to the traffic police data, there was a nearly 50% reduction of road traffic accidents during the first six months in 2020 compared to that of 2019 [7]. Despite restricted mobility, during the second quarter of 2020, the reported number of tuberculosis and leptospirosis were increased by around two to 2.5 folds respectively. The reduction of the SIR from 1.21to 0.91 indicates the impact of mobility restriction on the potential chickenpox outbreak that started during the first quarter. These heterogeneous communicable disease reporting patterns which are compatible with the transmission dynamics of each disease support our observation of the continuous functioning of communicable diseases surveillance system even during the lockdown period. Therefore, the observed reduction of SIR of dengue from 0.9 to 0.34 (2.6-fold reduction) may largely be due to altered transmission dynamics with restricted human mobility. Even when an adjustment is made to this 2.6-fold reduction of SIR of dengue assuming the fact that a 1.3-fold reduction of hospital admission was solely due to reduced health care seeking behaviour (which is the worst-case scenario), still there is another 1.3-fold reduction of SIR remaining.

We further compared the ratio between dengue fever (DF) to severe dengue (dengue haemorrhagic fever /DHF) reported in one of the hospitals draining the greatest number of dengue patients in Kalutara district using indoor mortality and morbidity data from 2015 to 2020. We observed that the DF to DHF ratio during 2020 appeared to be well within any random variability the previous years (Table C).

**Table C:** **Comparison between hospital admission due to dengue fever and severe dengue fever (dengue haemorrhagic fever) in Panadura hospital in Kalutara district from the year 2015 to 2020.**

| **Year** | **Dengue Fever (DF)** | **Dengue Hemorrhagic fever (DHF)** | **DF to DHF ratio** |
| --- | --- | --- | --- |
| 2015 | 437 | 139 | 0.32 |
| 2016 | 707 | 287 | 0.41 |
| 2017 | 3167 | 1268 | 0.40 |
| 2018 | 876 | 251 | 0.29 |
| 2019 | 2060 | 651 | 0.32 |
| 2020 | 305 | 127 | 0.42 |

Reference:

1. Liyanage P, Tissera H, Sewe M, Quam M, Amarasinghe A, Palihawadana P, et al. A spatial hierarchical analysis of the temporal influences of the el niño-southern oscillation and weather on dengue in Kalutara District, Sri Lanka. Int J Environ Res Public Health. 2016;13(11).

2. Bernal JL, Cummins S, Gasparrini A. Interrupted time series regression for the evaluation of public health interventions: A tutorial. Int J Epidemiol. 2017;46(1):348–55.

3. Wood SN. Generalized additive models: An introduction with R, second edition [Internet]. Generalized Additive Models: An Introduction with R, Second Edition. Chapman and Hall/CRC; 2017 [cited 2020 Oct 31]. 1–476 p. Available from: https://www.taylorfrancis.com/books/9781498728348

4. Gasparrini A, Armstrong B, Kenward MG. Multivariate meta-analysis for non-linear and other multi-parameter associations. Stat Med. 2012;31(29):3821–39.

5. Huedo-Medina TB, Sánchez-Meca J, Marín-Martínez F, Botella J. Assessing heterogeneity in meta-analysis: Q statistic or I 2 Index? Psychol Methods [Internet]. 2006 Jun [cited 2021 Jan 2];11(2):193–206. Available from: https://pubmed.ncbi.nlm.nih.gov/16784338/

6. Ministry of Healh Sri Lanka. ANNUAL HEALTH BULLETIN 2018 Ministry of Health [Internet]. [cited 2021 Feb 25]. Available from: www.health.gov.lk

7. Farzan Z. 8880 motor accidents so far in 2020; 106 CCTV Cameras monitor traffic in Colombo [Internet]. www.newsfirst.lk. 2020 [cited 2021 Feb 6]. p. 1. Available from: https://www.newsfirst.lk/2020/06/15/8880-motor-accident-so-far-in-2020-106-cctv-cameras-monitor-traffic-in-colombo/
