# Supplementary material for: The impact of COVID–19 lockdown on dengue transmission in Sri Lanka; A natural experiment for understanding the influence of human mobility
Source: PLoS Negl Trop Dis. 2021 Jun 10;15(6):e0009420. doi: 10.1371/journal.pntd.0009420 (PMC8192006; doi:10.1371/journal.pntd.0009420)
Supplement: S1 Table — (DOCX) [file pntd.0009420.s003.docx]

**Supporting Information (S1 Table)**

**Comparison between the quarterly distribution of dengue incidence by administrative districts.** Reported dengue cases per 100,000 population in each district during the first (January to May) and second (April to June) quarters in 2020 are compared to the five-year average (from 2015 to 2019) for the respective quarters in each district. SIR Q1 and SIR Q2 represent the standardized incidence ratios for the first and second quarters respectively.

| Province and District | **Population** | **5-year average (1st Quarter)** | **5-year average (2nd Quarter)** | **2020 (1st Quarter)** | **2020 (2nd Quarter)** | **SIR Q1** | **SIR Q2** |
| --- | --- | --- | --- | --- | --- | --- | --- |
| Colombo | 2324349 | 175·7 | 177·3 | 115·9 | 21·3 | 0·66 | 0·12 |
| Gampaha | 2304833 | 95 | 128·9 | 70·7 | 13·6 | 0·74 | 0·11 |
| Kalutara | 1221948 | 83·8 | 99·2 | 75·4 | 33·3 | 0·9 | 0·34 |
| Batticaloa | 526567 | 185 | 202·6 | 372·2 | 45·4 | 2·01 | 0·22 |
| Rathnapura | 1088007 | 60.3 | 111.7 | 52.5 | 61.4 | 0.9 | 0.5 |
| Trincomalee | 379541 | 228·3 | 118·9 | 570·2 | 22·7 | 2·50 | 0·19 |
| Jaffna | 583882 | 231·7 | 84·4 | 292 | 35·6 | 1·26 | 0·42 |
| Vavuniya | 172115 | 87·3 | 56 | 131·3 | 7·6 | 1·50 | 0·13 |
| Kilinochchi | 113510 | 72·6 | 39·5 | 90·7 | 11·5 | 1·25 | 0·29 |
| Mannar | 99570 | 102·4 | 49·6 | 117·5 | 5 | 1·15 | 0·1 |
| Mulativu | 92238 | 72 | 44·5 | 81·3 | 3·3 | 1·13 | 0·07 |
| Kandy | 1375382 | 53 | 90·4 | 78·4 | 52 | 1·48 | 0·58 |
| Matale | 484531 | 51·1 | 52·8 | 88·7 | 10·9 | 1·74 | 0·21 |
| Nuwara Eliya | 711644 | 11 | 10·9 | 17 | 2 | 1·55 | 0·18 |
| Galle | 1063334 | 65·8 | 76·2 | 89·1 | 14 | 1·35 | 0·18 |
| Hambantota | 599903 | 63·2 | 55 | 43 | 5·3 | 0·68 | 0·1 |
| Matara | 814048 | 58·9 | 58·0 | 43·1 | 0·1 | 0·73 | 0·01 |
| Kurunegala | 1618465 | 48·4 | 68·8 | 40·2 | 5·2 | 0·83 | 0·08 |
| Puttalam | 762396 | 64·7 | 63·9 | 45 | 6·6 | 0·7 | 0·1 |
| Badulla | 815405 | 32·5 | 32·1 | 43·4 | 6·6 | 1·34 | 0·21 |
| Moneragala | 451058 | 46·2 | 50·9 | 0 | 0 | 0 | 0 |
| Anuradhapura | 860575 | 33·7 | 30·8 | 37·4 | 4·4 | 1·11 | 0·14 |
| Polonnaruwa | 406088 | 30·8 | 39·3 | 44·6 | 7·1 | 1·45 | 0·18 |
