# Supplementary material for: The impact of COVID–19 lockdown on dengue transmission in Sri Lanka; A natural experiment for understanding the influence of human mobility
Source: PLoS Negl Trop Dis. 2021 Jun 10;15(6):e0009420. doi: 10.1371/journal.pntd.0009420 (PMC8192006; doi:10.1371/journal.pntd.0009420)
Supplement: S2 Table — (DOCX) [file pntd.0009420.s004.docx]

**Supporting Information (S2 Table)**

**Impact of lockdown intervention by administrative districts as estimated by the ITS model.** The relative risk and the percentage reduction with respective 95% confidence intervals as estimated by the first stage model are given by districts. The districts belong to the wet zone, dry zone, and western province are arranged separately. The meta estimates for the wet zone, dry zone, and western province are provided.

|  | **Relative risk (95% CI)** | **Percentage Reduction (95% CI)** |
| --- | --- | --- |
| **Wet Zone** |  |  |
| Colombo | 0·13 (0·02 to 0·74) | 87·47 (26·47 to 97·86) |
| Galle | 0·09 (0·02 to 0·38) | 91·28 (62·18 to 97·99) |
| Gampaha | 0·09 (0·01 to 0·69) | 91·16 (31·25 to 98·86) |
| Kalutara | 0·25 (0·08 to 0·74) | 75·48 (25·77 to 91·90) |
| Kandy | 0·25 (0·07 to 0·93) | 74·50 (7·09 to 93·00) |
| Kegalle | 0·21 (0·05 to 0·90) | 78·91 (9·51 to 95·08) |
| Matara | 0·002 (0·00 to 433·51) | 99·87 (-43250·67 to 100·00) |
| Nuwara Eliya | 0·07 (0·01 to 0·39) | 93·02 (60·78 to 98·76) |
| Ratnapura | 0·44 (0·16 to 1·21) | 56·14 (-20·93 to 84·09) |
| **Meta Estimate** | **0·17 (0·09 to 0·30)** | **83·34 (70·12 to 90·71)** |
| **Dry Zone** |  |  |
| Jaffna | 0·26 (0·05 to 1·29) | 73·56 (-29·31 to 94·59) |
| Kilinochchi | 0·14 (0·02 to 0·95) | 86·13 (4·85 to 97·98) |
| Mullaitivu | 0·05 (0·004 to 0·65) | 94·76 (35·25 to 99·58) |
| Mannar | 0·11 (0·004 to 2·94) | 88·91 (-194·31 to 99·58) |
| Vavuniya | 0·06 (0·005 to 0·76) | 93·85 (24·03 to 99·50) |
| Trincomalee | 0·06 (0·004 to 0·99) | 93·54 (1·44 to 99·58) |
| Anuradhapura | 0·08 (0·012 to 0·50) | 92·32 (50·49 to 98·81) |
| Polonnaruwa | 0·08 (0·020 to 0·34) | 91·79 (66·41 to 97·99) |
| Batticaloa | 0·11 (0·029 to 0·42) | 89·10 (58·41 to 97·14) |
| Ampara | 0·04 (0·011 to 0·13) | 96·33 (87·27 to 98·94) |
| Hambantota | 0·10 (0·023 to 0·47) | 89·61 (52·62 to 97·72) |
| Moneragala | 0·79 (0·000 to infinity) | 21·49 (100·00 to infinity) |
| **Meta Estimate** | **0·09 (0·05 to 0·15)** | **91·00 (85·00 to 95·00)** |
| **Western Province** |  |  |
| Colombo | 0·13 (0·02 to 0·74) | 87·47 (26·47 to 97·86) |
| Gampaha | 0·09 (0·01 to 0·69) | 91·16 (31·25 to 98·86) |
| Kalutara | 0·25 (0·08 to 0·74) | 75·48 (25·77 to 91·90) |
| **Meta Estimate** | **0·16 (0·06 to 0·43)** | **84·00 (57·00 to 94·00)** |
